# Constrained Multiobjective Biogeography Optimization Algorithm

**DOI:** 10.1155/2014/232714

**Published:** 2014-05-26

**Authors:** Hongwei Mo, Zhidan Xu, Lifang Xu, Zhou Wu, Haiping Ma

**Affiliations:** ^1^Automation College, Harbin Engineering University, Harbin 150001, China; ^2^Institute of Basic Science, Harbin University of Commerce, Harbin 150028, China; ^3^Engineering Training Center, Harbin Engineering University, Harbin 150001, China; ^4^Department of Electrical, Electronic and Computer Engineering, University of Pretoria, Gauteng 0028, South Africa; ^5^Department of Electrical Engineering, Shaoxing University, Shaoxing, Zhejiang 312000, China

## Abstract

Multiobjective optimization involves minimizing or maximizing multiple objective functions subject to a set of constraints. In this study, a novel constrained multiobjective biogeography optimization algorithm (CMBOA) is proposed. It is the first biogeography optimization algorithm for constrained multiobjective optimization. In CMBOA, a disturbance migration operator is designed to generate diverse feasible individuals in order to promote the diversity of individuals on Pareto front. Infeasible individuals nearby feasible region are evolved to feasibility by recombining with their nearest nondominated feasible individuals. The convergence of CMBOA is proved by using probability theory. The performance of CMBOA is evaluated on a set of 6 benchmark problems and experimental results show that the CMBOA performs better than or similar to the classical NSGA-II and IS-MOEA.

## 1. Introduction

In the domain of science and engineering, most of the problems are attributed to constrained multiobjective optimization problems (CMOPs), which need to optimize multiple conflicting objectives subject to various inequality and equality constraints. So the algorithms of solving CMOPs have to search the set of nondominated feasible solutions fulfilling all constraints. It is desirable that those gained solutions can approximate the true Pareto front with better diversity and even distribution. Evolutionary algorithms (EAs) are population-based search algorithms and can find multiple optimal solutions in one single run, and they are suitable to solve multiobjective problems (MOPs). But for the specific application of solving CMOPs, we find that most of the existing constrained multiobjective EAs (MOEAs) cannot effectively exploit the population because their obtained convergence and diversity are not acceptable.

Biogeography-based optimization (BBO) algorithm is a population-based search algorithm [1, 2], which had been applied to solve single objective optimization problems (SOPs) and some engineering problems [3–5]. In the aspect of MOPs, Ma et al. decomposed multiobjective optimization problems into several related subproblems and used parallel BBO to optimize each subproblem [6]. We successfully improved BBO for MOPs, which had proved that the migration strategy of BBO is effective for solving MOPs [7, 8]. In view of good population exploiting ability of BBO, in this study, we propose a novel constrained multiobjective biogeography optimization algorithm (CMBOA) for the first time and analyze its convergence by the probability theory.

The proposed CMBOA includes the following features. First, the individuals are classified into the feasible and infeasible ones based on their constraint violation. Second, feasible individuals are evaluated by combining their objective functions value and crowded distance. Infeasible individuals are evaluated by combining their constraint violation and Euclidean distance from the nearest nondominated feasible individual. Third, a new migration operator with additional disturbance is designed to generate diverse feasible solutions. And infeasible solutions nearby feasible regions are recombined with their nearest nondominated feasible solutions to evolve towards feasibility.

In rest of the paper, reviews of multiobjective evolutionary algorithms (MOEAs) for CMOPs are given in Section 2, and basic conception of CMOPs, the review of CMOPs, and the BBO are briefly introduced. The CMBOA is proposed in Section 3. In Section 4, compared with the classical algorithms on benchmark CMOPs, simulation results on CMBOA are analyzed and discussed. At last, conclusions are drawn in Section 5.

## 2. Related Background

### 2.1. Problem Statement

The aim of the constrained multiobjective optimization problems (constrained MOPs) is to find multiple nondominated solutions under constraints. If these nondominated solutions are uniformly distributed and widely spread along the Pareto front, their quality is better. Without the loss of generality, we consider the minimization of CMOPs, which can be defined as follows:

(1) min⁡ y=F(x)=[f1(x),f2(x),…,fm(x)]s.t gi(x)≤0, i=1,2,…,qhj(x)=0, j=q+1,q+2,…,lx=(x1,x2,…,xn)ximin⁡≤xi≤ximax⁡, i=1,2,…,n,

where **x** = (*x*_1_, *x*_2_,…, *x*_*n*_) ∈ *R* ^*n*^ is a decision vector with *n* decision variables. **y** = (*f*_1_, *f*_2_,…, *f*_*m*_) ∈ *R* ^*m*^ is an objective vector with *m* objects. Each dimension variable of the decision space is bounded by its upper bound *x*_*i*_^max⁡^ and lower bound *x*_*i*_^min⁡^. *g*_*i*_(**x**) and *h*_*j*_(**x**) are inequality constraints and equality constraint, respectively. The equality constraints generally should be transformed into inequality form and combined with other inequality constraints as follows:

(2) $$\begin{array}{c} G_{j}\left(x\right)=\{\begin{array}{c} \max\left\{g_{i}\left(x\right),0\right),\\ \max\left\{\;|\;h_{j}\left(x\right)-\delta ,0\right), \end{array} \end{array}$$

where *i* = 1,2,…, *q*, *j* = *q* + 1, *q* + 2,…, *l*, and *δ* is a tolerance parameter for the equality constraint. In this paper, only CMOPs with inequality constraints are considered. Constraint violation function of a solution *x* is defined as follows:

(3) $$\begin{array}{c} v\left(x\right)=\overset{n}{\underset{i=1}{\sum }}{\left(G_{i}\left(x\right)\right)}^{2}, \end{array}$$

where *v*(*x*) ≥ 0. If *v*(*x*) > 0, then *x* is an infeasible solution; otherwise, it is a feasible solution. By the degree of constraint violation, infeasible solutions can be compared with another one. For feasible solutions, Pareto domination is defined as follows, which is applied to evaluate their fitness.

**Definition 1 (Pareto domination solution)**

Let *x*, *y* ∈ *R* ^*n*^, and a solution vector *x* is said to dominate a solution *y* and is denoted by *x*≺*y* if

(4) $$\begin{array}{c} \forall i\in \left\{1,2,\ldots ,m\right\}:f_{i}\left(x\right)\leq f_{i}\left(y\right),\\ \exists j\in \left\{1,2,\ldots ,m\right\}:f_{j}\left(x\right)\leq f_{j}\left(y\right). \end{array}$$

### 2.2. Reviews of CMOEA

Most of MOEAs are proposed for solving unconstrained multiobjective optimization [9]. According to different constraint handling methods adopted in MOEAs, the existing constrained multiobjective evolutionary algorithms (CMOEAs) can be categorized into five main groups.

The first group adopts the constraint handling techniques applied for single objective constraint optimization [10–12]. Geng et al. proposed a constrained evolutionary multiobjective optimization with infeasible elitists and stochastic ranking selection (IS-MOEA) [10]. The algorithm conserves infeasible elitists that acts as bridges connecting disconnected feasible regions, and stochastic ranking is adopted to balance objectives and constraints. IS-MOEA especially obtains improvement on the problems with two or more disconnected feasible regions.

The second group uses the basic mechanism of MOEAs and handles constraints by optimizing them as additional objectives. Mezura-Montes and Coello put forward a naïve method to solve CMOPs by ignoring infeasible solutions [13]. The algorithm is easy to implement, but when feasible regions are small and surrounded by infeasible solutions, it is difficult to find feasible solutions.

The third group is based on ranking of priority of the feasible and infeasible solutions [14–16]. Fonseca and Fleming proposed a unified approach for multiobjective optimization and multiple constraint handling [14]. Their algorithm handled constraints by assigning high priority to constraints and low priority to objective functions, when focusing on search of feasible solutions. Srinivas and Deb proposed a constrained multiobjective algorithm, in which constrained dominating relation of individuals is defined [16]. In this algorithm, all feasible solutions dominate all infeasible ones. Feasible solutions are sorted by their Pareto dominating relations and infeasible solutions are sorted based on their constraint violation. The algorithm can gain better performance but unfortunately it ignored the contribution of infeasible solutions to the Pareto front.

The forth group uses repair scheme to reproduce feasible solutions or less violated solutions from the original infeasible solutions [17–19]. Jimenez et al. proposed the evolutionary algorithm of nondominated sorting with radial slots (ENORA) [17], which employs the min.-max. formulation for constraint handling. Feasible individuals evolve toward optimality, while infeasible individuals evolve toward feasibility. Harada et al. proposed Pareto descent repair (PDR) operator that searches feasible solutions out of infeasible individuals in the constraint function space [19].

The fifth group designs new mechanisms to evolve feasible solutions towards Pareto front and evolve the infeasible solutions towards feasible regions [20–23]. Ray et al. suggested using three different nondominated rankings of the population [20]. The first ranking is performed by using the objective function values; the second is performed by using different constraints; and the last ranking is based on the combination of all objective functions and constraints. Depending on these rankings, the algorithm performs according to the predefined rules. Chafekar et al. proposed two novel approaches for solving constrained multiobjective optimization problems [21]. One method called objective exchange genetic algorithm of design optimization (OEGADO) runs several GAs concurrently with each GA optimizing one objective and exchanging information about its objective with others. Another called objective switching genetic algorithm for design optimization (OSGADO) runs each objective sequentially with a common population for all objectives. Deb proposed GA's population-based approach that does not require any penalty parameter. Once sufficient feasible solutions are found, a niching method (along with a controlled mutation operator) is used to maintain diversity among feasible solutions [23].

### 2.3. Biogeography-Based Optimization (BBO)

Biogeography is the science of the geographical distribution of biological organisms. In BBO, each problem solution is considered as a “habitat” with habitat survival index (HSI), which is similar to the fitness of EAs to evaluate an individual. High HSI habitats share their features with low HSI habitats. The process of sharing good features among solutions is denoted as migration. BBO adopts the migration strategy to share information among solutions. Good individuals' information can be conserved during the evolutionary process to ensure the population convergence. A mutation operator is used to generate diverse solutions to promote the diversity of the population. The detailed operations are described as follows.

Suppose that the species number of each individual *i* is *S*_*i*_, and then its immigration rate *λ*_*i*_ and emigration rate *μ*_*i*_ can be calculated as follows [1]:

(5) $$\begin{array}{c} \lambda_{i}=I\left(1-\frac{S_{i}}{S_{\max}}\right),\\ \mu_{i}=\frac{ES_{i}}{S_{\max}}, \end{array}$$

where *S*_max⁡_ is the most species number of all habitats. *I* and *E* represent the maximization of immigration rate and emigration rate, respectively. In migration operator, the individuals' immigration rate and emigration rate are used to decide whether a solution should share its feature value with the other solutions. A better solution has a higher immigration rate and a lower emigration rate. By the migration, the solutions with high emigration rate tend to share their information with those with high immigration rate. Solutions with high immigration rate accept a lot of features from solutions with high emigration rate. With the aid of migration, BBO shows good exploitation ability in the search space.

Consider that species number change with species migrating; the probability *P*_*s*_ that the habitat contains exactly *S* species can be calculated using the following differential equation:

(6) $$\begin{array}{c} \overset{.}{P_{s}}=\{\begin{array}{c} -\left(\lambda_{s}+\mu_{s}\right)P_{s}+\mu_{s+1}P_{s+1},\\ S=0,\\ -\left(\lambda_{s}+\mu_{s}\right)P_{s}+\lambda_{s-1}P_{s-1}+\mu_{s+1}P_{s+1},\\ 1\leq S<S_{\max}-1\\ -\left(\lambda_{s}+\mu_{s}\right)P_{s}+\lambda_{s-1}P_{s-1},\\ S=S_{\max}. \end{array} \end{array}$$

Then the mutation rate *m*_*i*_ is defined as [1]

(7) $$\begin{array}{c} m_{i}=P_{\text{mute}}\left(1-\frac{P_{i}}{P_{\max}}\right), \end{array}$$

where *P*_mute_ is a predefined parameter, *P*_*i*_ is calculated according to (6), and *P*_max⁡_ = max⁡_1≤*i*≤*N*_⁡{*P*_*i*_}. The mutation operator is implemented based on *m*_*i*_. A solution with low probability *P*_*i*_ is likely to mutate other solutions. Conversely, some solutions with high *P*_*i*_ have very little chance to mutate. By the mutation operator, the diverse solutions are produced. The detailed operator on migration and mutation can refer to [1].

## 3. The Proposed Constrained Multiobjective Biogeography-Based Optimization Algorithm

### 3.1. CMBOA Description

In CMBOA, infeasible solutions recombine with nondominated feasible individuals and evolve towards feasibility. Firstly, the initial population is produced stochastically, and then the population is classified into the feasible and infeasible ones based on each individual's constraint violation. Secondly, depending on whether the feasible population is empty or not, infeasible population will adopt two types of operators. If feasible population is empty, infeasible population will implement differential evolution operator until feasible individuals present; otherwise, infeasible solutions nearby feasible regions recombine with their nearest nondominated feasible solutions to obtain feasibility. Diverse nondominated feasible solutions are generated from feasible individuals by applying the novel migration operator. With the increasing of nondominated feasible solutions, update operator is used to limit their number and ensure their even distribution. Both the feasible and infeasible solutions are combined in an external archive. The proposed CMBOA is described as Algorithm 1.

The procedure of CMBOA is described as follows.

**Step 1 (initialization)**

Initialize the iterative number *t* = 1; the size of feasible elitist and infeasible elitist archive are *N*_1_ and *N*_2_, respectively. Generate randomly the initial population *A*(*t*) with *N*(*t*) individuals; that is *A*(*t*) = {*a*_1_(*t*), *a*_2_(*t*),…, *a*_*N*(*t*)_(*t*)}, the external archive *M*(*t*) = Φ.

**Step 2**

Update the external archive.

*Step 2* *.1*. Divide the combination populations *A*(*t*) ∪ *M*(*t*) into the feasible and infeasible ones.

Computing the constraint violation of the individuals in *A*(*t*) ∪ *M*(*t*) according to (3), we have

(8) $$\begin{array}{c} v\left(A\left(t\right)\cup M\left(t\right)\right)=\left\{v\left(a_{1}\left(t\right)\right),v\left(a_{2}\left(t\right)\right),\ldots ,v\left(a_{N(t)}\left(t\right)\right)\right\}. \end{array}$$

Depending on whether the value of *v*(*a*_*i*_(*t*)) is zero or not, the population *A*(*t*) ∪ *M*(*t*) is divided into the feasible subpopulation *P*(*t*):

(9) $$\begin{array}{c} P\left(t\right)=\left\{p_{1}\left(t\right),p_{2}\left(t\right),\ldots ,p_{N_{f}(t)}\left(t\right)\right\} \end{array}$$

and the infeasible subpopulation *Q*(*t*):

(10) $$\begin{array}{c} Q\left(t\right)=\left\{q_{1}\left(t\right),q_{2}\left(t\right),\ldots ,q_{N_{\text{if}}(t)}\left(t\right)\right\}. \end{array}$$

Note that *N*_*f*_(*t*) + *N*_if_(*t*) = *N*(*t*).

*Step 2.2 (elitist feasible and infeasible archive)*. According to Definition 1, identify nondominated individuals of *P*(*t*) to form the temporary set *P*′(*t*):

(11) $$\begin{array}{c} P^{\prime }\left(t\right)=\left\{p_{1}^{\prime }\left(t\right),p_{2}^{\prime }\left(t\right),\ldots ,p_{\text{non}\left(t\right)}^{\prime }\left(t\right)\right\}. \end{array}$$

If the size of *P*′(*t*) is smaller than the predefined size *N*_1_, let *P*(*t*) = *P*′(*t*). Otherwise, the crowding distance *I*_*i*.cd_(*p*_*i*_′(*t*)) of individual *p*_*i*_′(*t*), 1 ≤ *i* ≤ non is computed as follows [24]:

(12) Icd(pi′(t))=(f1(pi′(t))−f1(pi−1′(t))) +(f2(pi′(t))−f2(pi−1′(t))),

where *f*_*k*_(*p*_*i*_′(*t*)) denotes the* k*th objective function value of individual *p*_*i*_′(*t*), 1 ≤ *i* ≤ non. According to the sequencing of crowding distance, select *N*_1_ largest crowding distance individuals from *P*′(*t*) to form the elitist feasible archive *P*(*t*):

(13) P(t)=Td(P′(t))=Td{p1′(t),p2′(t),…,pnon(t)′(t)}={p1(t),p2(t),…,pN1(t)}.

For the infeasible population *Q*(*t*), if its size is smaller than the predefined size *N*_2_, then *Q*(*t*) keeps invariable. Otherwise, the fitness of its individual *q*_*i*_(*t*) is calculated as follows:

(14) $$\begin{array}{c} \text{fit}\left(q_{i}\left(t\right)\right)=\{\begin{array}{cc} \left(1-\gamma \right)v\left(q_{i}\left(t\right)\right)+\gamma d\left(q_{i}\left(t\right)\right), & \gamma >0,\\ v\left(q_{i}\left(t\right)\right), & \gamma =0, \end{array} \end{array}$$

where *γ* is the proportion of nondominated feasible individuals in current population, *v*(*q*_*i*_(*t*)) is constraint violation of the individual *q*_*i*_(*t*), and *d*(*q*_*i*_(*t*)) denotes its Euclidean distance away from the nearest nondominated feasible solution. And then the proceeding *N*_2_ individuals with small fitness from *Q*(*t*) are conserved in elitist infeasible archive *Q*(*t*).

*Step 2.3 (formation of the archive)*. Combine elitist feasible archive *P*(*t*) and elitist infeasible archive *Q*(*t*) to gain the archive *M*(*t*):

(15) M(t)=P(t)∪Q(t)={p1(t),p2(t),…,pN1(t),q1(t),q2(t),…,qN2(t)}={m1(t),m2(t),…,mN1(t),…,mN1+N2(t)}.

If *t* ≥ *g*_max⁡_ is satisfied, export *P*(*t*) as the output of the algorithm and the algorithm stops; otherwise, *t* = *t* + 1 and go to Step 3.

**Step 3**

Generate the offspring population.

*Step 3.1 (operation on feasible solutions)*. In CMBOA, when there are no feasible solutions in the current population, that is, *p*(*t*) = Φ, we use the mutation operator of differential evolution to produce feasible individuals [25]. That is, three individuals *q*_*r*1_(*t*), *q*_*r*2_(*t*), and *q*_*r*3_(*t*) are selected randomly from *Q*(*t*) and the mutation operator is performed as (16) until feasible solutions set *p*(*t*) is not empty:

(16) $$\begin{array}{c} p_{i,j}\left(t\right)=q_{r1,j}\left(t\right)+\eta \left(q_{r2,j}\left(t\right)-q_{r3,j}\left(t\right)\right), \end{array}$$

where *η* is a mutation constant and is a random number in the region (0,1). Otherwise, go to the next step.

*Step 3.2 (selection operation)*. For feasible population *P*(*t*), in order to ensure its convergence and even distribution, we define the fitness value of each individual by combining the nondominated rank and crowed distance of each individual *p*_*i*_(*t*), 1 ≤ *i* ≤ *N*_1_ as

(17) $$\begin{array}{c} \text{fit}\left(p_{i}\left(t\right)\right)=\frac{\left(1-\gamma \right)}{c_{ik}\left(p_{i}\left(t\right)\right)}+\gamma I_{\text{cd}}\left(p_{i}\left(t\right)\right), \end{array}$$

where *I*_cd_(*p*_*i*_(*t*)) and *c*_*ik*_(*p*_*i*_(*t*)) denote the individual *p*_*i*_(*t*)'s crowed distance and nondominated rank, respectively, and *γ* is defined in (14). By this fitness, when the number of nondominated feasible solutions is small, individuals with lower ranks have high fitness so that they have more chance to be selected. With the number of nondominated feasible solutions increasing, more individuals with large crowded distance are selected with high probability.

Perform tournament selection operator *T*_*S*_ on *P*(*t*) to form the breeding pool *D*(*t*):

(18) D(t)=TS(P(t))=TS{p1(t),p2(t),…,pN1(t)}={d1(t),d2(t),…,dN1(t)}.

*Step 3.3 (migration operation)*. The original migration operator of BBO has good exploitation ability of the population, but it is designed for the integer encoded individuals and single optimization problem. For continuous MOPs, the migration operator cannot ensure to produce the diverse solutions. So we propose a new migration operator. During the process of species migration, an individual is often affected by the other individuals. So we introduce a disturbance term in the migration operation to promote the diversity of the population. The detail process is shown in Algorithm 2.

In Algorithm 2, the disturbance factor *ω*(*t*) is defined as

(19) $$\begin{array}{c} \omega \left(t\right)=\frac{4}{5}\left(1-\frac{1}{1+e^{-0.1(t-g_{\max}/2)}}\right), \end{array}$$

where *d*_*i*,*j*_(*t*) is the *j*th variable of the individual *d*_*i*_(*t*), *g*_max⁡_ denotes the maximum iteration number, and *t* is the number of iteration at current generation. The amplitude of disturbance factor *ω*(*t*) decreases constantly with the increasing of generation *t*. At the beginning, large disturbance makes the population explore a wide region in decision space. Diverse solutions will be generated to promote the diversity of population because of difference of *ω*(*t*). At the end, a small disturbance is used to exploit effectively the local regions to guarantee its convergence.

The migration operator *T*_*i*_ on the population *D*(*t*) is defined as

(20) P(t+1)=Ti(D(t))={Ti(d1(t)),Ti(d2(t)),…,Ti(dN1(t))}={p1(t),p2(t),…,pN1(t)}.

*Step 3.4 (crossover and mutation operation on the infeasible population)*. It had been noticed that infeasible solutions can contribute to the diversity of solutions on the Pareto front. When feasible solutions exist in the current population, an individual *q*_*r*1_(*t*) is selected randomly from *Q*(*t*) and recombined with the nearest individual *d*_*r*2_(*t*) of *D*(*t*). The crossover operator is described as

(21) $$\begin{array}{c} q_{i,j}\left(t+1\right)=\lambda q_{r1,j}\left(t\right)+\left(1-\lambda \right)d_{r2,j}\left(t\right), \end{array}$$

where *λ* is a recombination parameter in the region (0,1). By the operator, infeasible individuals nearby the feasible region will approximate the feasibility.

The above crossover operation *T*_*c*_ on *Q*(*t*) is

(22) Q(t+1)=Tc(Q(t))={Tc(q1(t)),Tc(q2(t)),…,Tc(qN2(t))}={q1(t+1),q2(t+1),…,qN2(t+1)}.

*Step 3.5*. Combine *P*(*t* + 1) and *Q*(*t* + 1) to obtain the offspring population *A*(*t* + 1); namely, *A*(*t* + 1) = *P*(*t* + 1) ∪ *Q*(*t* + 1).

**Step 4**

If the stopping criteria is not satisfied, *t* = *t* + 1 and return to Step 2.

### 3.2. Time Complexity Analysis of CMBOA

The objectives of optimization problem are *m*, the size of population is *N*, the size of feasible archive is *N*_1_, the size of infeasible archive is *N*_2_, and the maximum of iterative times is *g*_max⁡_. Time complexity for computation of constraint violation is *O*(*N*). For migration and mutation operators on feasible individuals, its time complexity is *O*(*N*_1_), while time complexity for crossover operator on infeasible individuals is *O*(*N*_2_); time complexity for updating of feasible archive is *O*(*m*(2*N*_1_ + *N*_2_)^2^), updating of infeasible archive is *O*(*m*(*N*_1_ + 2*N*_2_)^2^), and then the worst time complexity of CMBOA is

(23) O(N)+O(N1)+O(N2)+O(m(2N1+N2)2)  +O(m(N1+2N2)2)=O(m(2N1+N2)2).

### 3.3. Convergence Analysis of CMBOA

According to the description of CMBOA, it can be considered as an evolution Markov chain:

(24) $$\begin{array}{c} A\left(t\right)\overset{T_{d}}{⟶}P\left(t\right)\overset{T_{s}}{⟶}D\left(t\right)\overset{T_{i}}{⟶}P\left(t+1\right)⟶A\left(t+1\right). \end{array}$$

Let *S* be feasible solution space, *S* ≤ *N* represents a state space composed of populations whose size is not more than *N*, and *s*_*i*_ denotes the* i*th state in state space. *A*_*t*_^*i*^ denotes that the population *A*(*t*) is in the state *s*_*i*_, and *p*(*P*_*t*_^*j*^ | *A*_*t*_^*i*^) means the transformation probability from *A*_*t*_^*i*^ to *P*_*t*_^*j*^. According to the description of CMBOA, we know that the series {*A*_*t*_}_*t*≥1_ is an inhomogeneous Markov chain [26]. By using probability theory, the convergence of CMBOA is analyzed as follows.

**Lemma 2**

There exists 0 < *δ*_1_ < 1, s.t. *p*(*P*_*t*+1_^*e*^ | *D*_*t*_^*d*^) ≥ *δ*_1_.

**Proof**

(25) p(Pt+1e ∣ Dtd)=∏x∈Dt,y∈Pt+1p(x,y)=∏x∈Dt,y∈Pt+1λxk(x,y)(1−λx)n−k(x,y)≥min⁡(λmin⁡,1−λmax⁡)N1=δ1,

where *α* = min⁡_*x*∈*D*_*t*__⁡{*λ*_*x*_}, *β* = max⁡_*x*∈*D*_*t*__⁡{*λ*_*x*_}, and *k*(*x*, *y*) = ∑_*x*_*i*_≠*y*_*i*_,1≤*i*≤*n*_sgn⁡|*x*_*i*_ − *y*_*i*_|.

**Lemma 3**

There exists 0 < *δ*_2_ < 1, s.t. *p*(*A*_*t*+1_^*j*^ | *P*_*t*+1_^*e*^) ≥ *δ*_2_.

**Proof**

(26) p(At+1j ∣ Pt+1e)=|Mf(Pt+1∪Qt+1∪Mt+1,≺)∩Mf(S,≺)||Pt+1∪Qt+1∪Mt+1|≥(M∗2(N1+N2))2N1=δ2,

where M* = |*M*_*f*_(*P*_*t*+1_ ∪ *Q*_*t*+1_ ∪ *M*_*t*+1_, ≺)∩*M*_*f*_(*S*, ≺)|  and *M*_*f*_(*P*_*t*+1_ ∪ *Q*_*t*+1_ ∪ *M*_*t*+1_, ≺) denotes the nondominated feasible solutions of the population *P*_*t*+1_ ∪ *Q*_*t*+1_ ∪ *M*_*t*+1_.

**Lemma 4**

Let *M*_*f*_(*s*, ≺) be the nondominated feasible solutions set; if *s*_*i*_∩*M*_*f*_(*s*, ≺) = Φ and *s*_*j*_∩*M*_*f*_(*s*, ≺) ≠ Φ, then there exists 0 < *δ* < 1, s.t.  *p*(*A*_*t*+1_^*j*^ | *A*_*t*_^*i*^) ≥ *δ*.

**Proof**

Using K-C equation, we can obtain

(27) p(At+1j ∣ Ati)=∑sd∑,se∈S≤2(N1+N2)p(Dtd ∣ Ati)p(Pt+1e ∣ Dtd)×p(At+1j ∣ Pt+1e).

Note that *p*(*D*_*t*_^*d*^ | *A*_*t*_^*i*^) = 1; based on Lemmas 2 and 3, we derive

(28) $$\begin{array}{c} p\left(A_{t+1}^{j}\;|\;A_{t}^{i}\right)\geq \delta_{1}\delta_{2}\equiv \delta . \end{array}$$

**Theorem 5**

CMBOA is weakly convergent for any initial population distribution; that is,

(29) $$\begin{array}{c} \underset{t\to \infty }{\lim}p\left(A_{t+1}\cap M_{f}\left(s,≺\right)=\Phi \right)=0. \end{array}$$

**Proof**

If *s*_*i*_∩*M*_*f*_(*s*, ≺) = Φ, then

(30) $$\begin{array}{c} p\left(A_{t+1}\cap M_{f}\left(s,≺\right)=\Phi \;|\;A_{t}^{i}\right)\leq 1-\delta . \end{array}$$

If *s*_*i*_∩*M*_*f*_(*s*, ≺) ≠ Φ, the archive *M*(*t*) is applied to conserve the elitist individuals; then

(31) $$\begin{array}{c} p\left(A_{t+1}\cap M_{f}\left(s,≺\right)=\Phi \;|\;A_{t}^{i}\right)=0. \end{array}$$

By (27) and (28), we can obtain

(32) p(At+1∩Mf(s,≺)=Φ) =∑si∩Mf(s,≺)=Φp(At+1∩Mf(s,≺)=Φ ∣ Ati)p(Ati)  +∑si∩Mf(s,≺)≠Φp(At+1∩Mf(s,≺)=Φ ∣ Ati)p(Ati) ≤(1−δ)t,lim⁡t→∞p(At+1∩Mf(s,≺)=Φ)=0.

Hence, CMBOA is weakly convergent for any initial population distribution.

## 4. Simulation Results

### 4.1. Experimental Setup

In order to test the validity of the proposed CMBOA, several benchmark functions with multiple features are selected including OSY [27], TNK [26], CONSTR [27], CTP1-CTP5 [28], CF1, CF2, CF4, and CF6 [29]. For OSY, its Pareto front is a concatenation of five regions and every region lies on the intersection of certain constraints; for TNK, its Pareto optimal solutions lie on a non-linear constraint surface; for CONSTR, its Pareto optimal set is concatenation of the constraint boundary and some parts of unconstrained Pareto optimal; for CTP serious functions, their Pareto optimal set is a collection of a number of discrete regions and most of solutions lie on non-linear constraint boundary. OSY, CONSTR, CTP1, CF4 and CF6 have continuous Pareto fronts, while the remaining ones have disjoint Pareto fronts (TNK, CTP2-CTP5, CF1, and CF2).

### 4.2. Performance Metrics

In this experiment, two performance metrics are selected to do quantitatively comparison.

*Cover Metric C [30]*. Suppose that* U* and* V* are two approximate Pareto optimal sets obtained by Algorithms 1 and 2:

(33) $$\begin{array}{c} C\left(U,V\right)=\frac{\left|\left\{u\in U;\exists v\in V:u\leq v\right\}\right|}{\left|V\right|}, \end{array}$$

where ≤ denotes the dominated or equal relation. The value *C*(*U*, *V*) = 1 represents that all individuals in *V* are weakly dominated by individuals in *U*. *C*(*U*, *V*) = 0 denotes no individuals in *V* which is weakly dominated by *U*. Note that *C*(*U*, *V*) ≠ 1 − *C*(*V*, *U*); hence two directions must be considered simultaneously.

*Hyper Volume HV [30]*. The indicator calculates the volume covered by all nondominated solutions in the objective space. For each solution *X*_*i*_, a hypercube *hv*_*i*_ is constructed with a predefined reference point and the solution *X*_*i*_ as the diagonal corners of the hypercube *hv*_*i*_. All hypercubes are found and HV is calculated as follows:

(34) $$\begin{array}{c} \text{HV}=\overset{\left|x\right|}{\underset{i=1}{⋃}}hv_{i}. \end{array}$$

The indicator is related to the approximation and diversity of the nondominated solution set. The higher the value of HV is, the better the diversity and approximation of solution set obtained is.

### 4.3. Performance of CMBOA on Benchmark Functions

#### 4.3.1. Test on Benchmark Functions

To demonstrate the effectiveness of the proposed CMBOA for CMOPs, 12 benchmark functions are chosen to show its validity. In the experiment, each individual is described as a real vector. The parameters of CMBOA are set as follows: population size = 100, feasible elitist maximum size *N*_1_ = 100, infeasible elitist maximum size *N*_2_ = 20, the maximum immigration rate and migration rate *E* = *I* = 1, the termination generation = 100, *F* is a random in the interval (0.2,0.8), and *CR* = 0.5.

**Figure 1:**
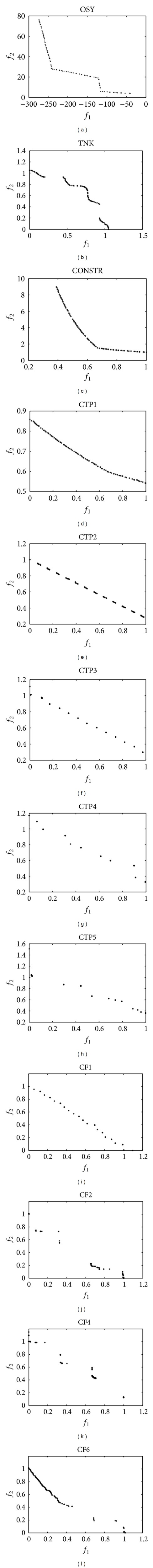
Final Pareto front for all test functions by the proposed algorithm CMBOA.

For all benchmark function, the Pareto fronts obtained by CMBOA are shown in Figure 1. From this figure, it can be seen that the Pareto optimal solutions obtained by CMBOA are very close to the true Pareto front for all benchmark functions. For most of benchmark functions, the solutions generated by the proposed algorithm can be distributed evenly on the true Pareto front except CF2, CF4, and CF6 because they have variable linkages.

#### 4.3.2. Comparison with Original Migration Operator

**Figure 2:**
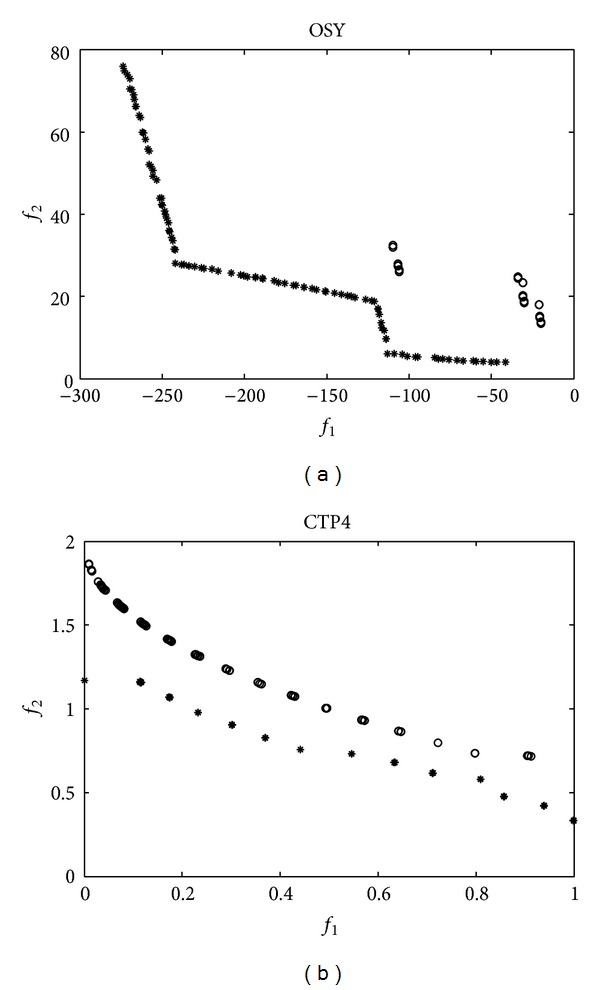
Final Pareto front for OSY and CTP4 under original and novel migration operator.

In order to demonstrate the effectiveness of the novel migration operation, OSY and CTP4 are selected. The Pareto fronts gained by the algorithms with original and novel migration operator are shown in Figure 2, where “∗” denotes the Pareto front gained by CMBOA with the novel migration and “o” is the ones gained by the algorithm with original migration operator. From Figure 2, it can be seen that the algorithm with original migration cannot converge to the true Pareto front for OSY and CTP4, and only few solutions are produced for OSY. However, CMBOA with the novel migration operator obtains good convergence and diversity for OSY and CTP4, which demonstrates that the novel migration operator is superior to the original migration operator for OSY and CTP4.

#### 4.3.3. Parameter Sensitivity Analysis

**Figure 3:**
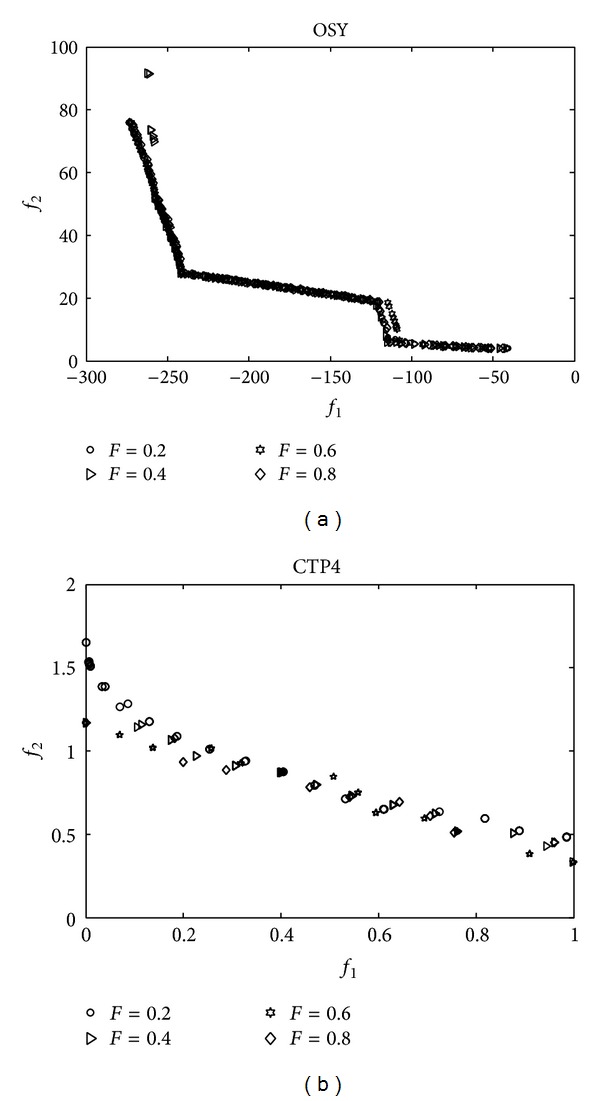
Final Pareto front for OSY and CTP4 under *F*(*t*) = 0.2,0.4,0.6,0.8.

The disturbance parameter *F*(*t*) is not tuned elaborately but is set as (20). In this section, to investigate the robustness of the disturbance parameter *F*(*t*), simulations with different settings *F*(*t*) = 0.2,0.4,0.6,0.8 are performed. Benchmark functions OSY and CTP4 are selected to test the sensitivity of *F*(*t*). The Pareto fronts gained under different *F*(*t*) settings are shown in Figure 3. From the results, it is observed that the algorithms with different *F*(*t*) settings can all converge to the true Pareto front for OSY and CTP4, which illustrates that the disturbance parameter *F*(*t*) is capable to perform consistently and effectively for OSY and CTP4. So the disturbance parameter *F*(*t*) is reliable and robust to produce better solutions.

### 4.4. Comparison with Other Algorithms

To show the superior performance of the proposed algorithm, it is compared with the most popular multiobjective algorithms including NSGA-II [24] and IS-MOEA [1]. For NSGAII, the parameters are set as population size = 100, crossover probability = 0.9, mutation probability = 1/*n*, SBX crossover parameter = 20, polynomial mutation parameter = 20, and the termination generation = 100. For IS-MOEA, the parameters are set as population size = 100, crossover probability = 0.9, mutation probability = 1/*n*, SBX crossover parameter = 20, polynomial mutation parameter = 20 comparison probability = 0.45, penalty parameters *ω*_*j*_ = 1, *β* = 1, and the termination generation = 100. For the proposed CMBOA, the parameters are set the same as the previous section. For all algorithms, 30 independent runs are conducted on each of the benchmark functions to get the statistical results in cover metric *C* and hypervolume HV. Their distribution of simulation results is shown in Tables 1–6.

In Table 1, if the value of* C* (CMBOA, NSGA-II) is larger than that of* C* (NSGA-II,CMBOA), it indicates that the proposed CMBOA has better convergence than NSGA-II; otherwise, it indicates that the proposed CMBOA is inferior to NSGA-II in term of convergence. From Table 1, it can be seen that for CONSTR, CF2, CF4, and CF6, and NSGA-II is better than CMBOA in term of convergence. However, for the other eight test functions, CMBOA has better convergence than NSGA-II. Wilcoxon rank-sum test is used to examine their difference [31] and the results are shown in Table 2. The alternative hypothesis is *p* ≤ *α* and *α* = 0.05. If *p* ≤ *α* is met, the algorithms have significant difference; otherwise, they have no difference. From Table 2, we can see that, compared with NSGA-II, CMBOA is significantly superior on OSY, CTP1, CTP3, CTP4, CTP5, CF1, and CF6 in converging close to Pareto front. In order to analyze their difference in convergence, the distribution of their cover metric values is studied by Wilcoxon rank-sum test which is summarized in Table 3. In Table 3, the CMBOA is significantly superior to IS-MOEA in most benchmark functions except TNK, CTP1, and CTP2 on convergence. In Table 4, we can see that, except TNK, CMBOA is superior to IS-MOEA in convergence, while IS-MOEA is better than CMBOA in TNK.

In order to evaluate the convergence and the diversity of solutions obtained by the proposed CMBOA, statistical results of hypervolume metric are summarized in Table 5. In this table, higher hypervolume value indicates that the algorithm has better diversity. From Table 5, it can be seen that CMBOA has better diversity than the other two algorithms for almost all test functions except CONSTR and CF4. From the variance of metric HV, we can see that CMBOA has the smallest variance which indicates that it is more reliable and robust than NSGA-II and IS-MOEA in producing better solutions. In order to analyze the distribution of HV value in further, its Wilcoxon rank-sum test value is summarized in Table 6. From Table 6, we can conclude that CMBOA is superior to NSGA-II and IS-MOEA in terms of the distribution and diversity of solutions except CONSTR, CTP2, and CF4. Experiment results above show that the CMBOA has competitive performance with NSGA-II and IS-MOEA in the terms of convergence and diversity.

## 5. Conclusions

In this paper, we propose a new constrained multiobjective biogeography-based optimization algorithm. A new disturbance migration operator is designed to generate diverse feasible solutions. Infeasible solutions nearby the feasible region are recombined with their nearest feasible ones to change the feasibility. Theoretically, the weak convergence of CMBOA is proved by the probability theory and its time complexity is analyzed. Experimentally, CMBOA is tested on several typical benchmark functions and compared with classical NSGA-II and IS-MOEA. The statistical results show that the proposed CMBOA is highly competitive in terms of convergence and diversity. In future work, we may improve CMBOA to obtain better performance on variable linkage problems.

## Figures and Tables

**Algorithm 1:**
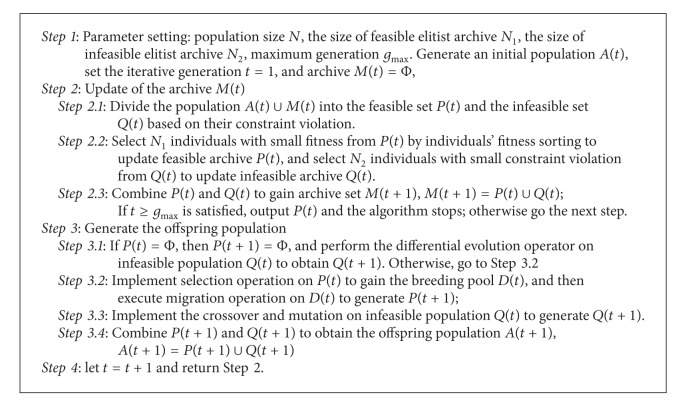
CMBOA.

**Algorithm 2:**
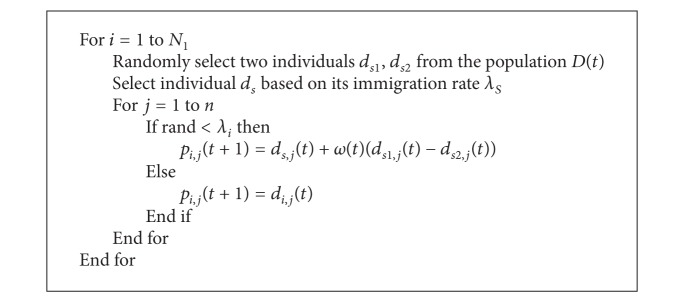
Disturbance migration operator *T*_*i*_.

**Table 1:** Mean and variance (Var.) of the cover metric on CMBOA and NSGA-II.

Algorithm	Benchmark functions					
	OSY	TNK	CONSTR	CTP1	CTP2	CTP3
*C*(CMBOA, NSGA-II)						
Mean	0.3003	0.2007	0.1320	0.2643	0.2690	0.7543
Var.	0.0324	0.0019	0.0012	0.0023	0.0039	0.0319
*C*(NSGA-II, CMBOA)						
Mean	0.1683	0.1940	0.1517	0.1270	0.2397	0.2567
Var.	0.0160	0.0011	0.0016	0.0026	0.0043	0.0262

Algorithm	Benchmark functions					
	CTP4	CTP5	CF1	CF2	CF4	CF6

*C*(CMBOA, NSGA-II)						
Mean	0.7507	0.6863	0.8403	0.1433	0.0860	0.2250
Var.	0.0540	0.0675	0.0138	0.0152	0.0209	0.0100
*C*(NSGA-II, CMBOA)						
Mean	0.1793	0.3150	0.1550	0.1683	0.2500	0.4093
Var.	0.0274	0.0296	0.0120	0.0080	0.0961	0.0135

**Table 2:** Wilcoxon rank-sum test on *C* value of CMBOA and NSGA-II.

	OSY	TNK	CONSTR	CTP1	CTP2	CTP3
(*C*(CMBOA, NSGA-II), *C*(NSGA-II, CMBOA))	**0.0020**	0.3937	0.0738	**5.6395e − 010**	0.1096	**4.3641e − 010**

	CTP4	CTP5	CF1	CF2	CF4	CF6

(*C*(CMBOA, NSGA-II), *C*(NSGA-II, CMBOA))	**6.3039e − 010**	**1.7176e − 006**	**3.1436e − 011**	0.1408	0.7492	**4.9194e − 007**

**Table 3:** Wilcoxon rank-sum test on *C* value of CMBOA and IS-MOEA.

	OSY	TNK	CONSTR	CTP1	CTP2	CTP3
(*C*(CMBOA, IS-MOEA), *C*(CMBOA, IS-MOEA))	**4.4824e − 012**	**2.8394e − 011**	**0.0010**	0.0685	**5.7712e − 009**	**0.0058**

	CTP4	CTP5	CF1	CF2	CF4	CF6

(*C*(CMBOA, IS-MOEA), *C*(CMBOA, IS-MOEA))	**3.2785e − 011**	**9.1907e − 009**	**1.8685e − 011**	**2.3115e − 010**	**1.1817e − 008**	**9.7713e − 012**

**Table 4:** Mean and variance (Var.) of the cover metric on CMBOA and IS-MOEA.

Algorithm	Benchmark functions					
	OSY	TNK	CONSTR	CTP1	CTP2	CTP3
*C*(CMBOA, IS-MOEA)						
Mean	0.9044	0.0997	0.1670	0.1823	0.1773	0.5976
Var.	0.0456	0.0010	0.0010	0.0048	0.0037	0.0318
*C*(IS-MOEA, CMBOA)						
Mean	0.0517	0.3093	0.1367	0.1523	0.3167	0.4710
Var.	0.0140	0.0029	0.0012	0.0017	0.0045	0.0249

Algorithm	Benchmark functions					
	CTP4	CTP5	CF1	CF2	CF4	CF6

*C*(CMBOA, IS-MOEA)						
Mean	0.7990	0.6701	0.9667	0.6591	0.6190	0.8472
Var.	0.0241	0.0334	0.0020	0.0680	0.0957	0.0305
*C*(IS-MOEA, CMBOA)						
Mean	0.1767	0.3223	0.0520	0.0787	0.0713	0.0143
Var.	0.0173	0.0133	0.0045	0.0109	0.0210	0.0011

**Table 5:** Mean and variance (Var.) of the hypervolume (HV) metric.

Algorithm	HV	Benchmark functions					
		OSY	TNK	CONST	CTP1	CTP2	CTP3
CMBOA	Mean	0.9835	0.998	0.9992	0.9995	0.9992	0.9949
	Var.	0.0032	3.0145*e* − 007	2.2481*e* − 007	9.3282*e* − 008	1.5011*e* − 007	1.3630*e* − 005
NSGA-II	Mean	0.9107	0.9965	0.9993	0.9944	0.9983	0.9763
	Var.	0.0162	5.7807*e* − 006	3.1099*e* − 007	4.1408*e* − 005	2.8101*e* − 006	0.0029
IS-MOEA	Mean	0.7576	0.9965	0.9992	0.9757	0.9579	0.9609
	Var.	0.0257	1.5731*e* − 004	2.8652*e* − 007	0.0021	0.0069	0.0011

Algorithm	HV	Benchmark Functions					
		CTP4	CTP5	CF1	CF2	CF4	CF6

CMBOA	Mean	0.9289	0.9190	0.9956	0.9549	0.8820	0.9535
	Var.	0.0015	0.0014	1.4497*e* − 005	0.0016	0.0077	8.3474*e* − 004
NSGA-II	Mean	0.8581	0.8180	0.9901	0.8971	0.9319	0.9445
	Var.	0.0185	0.0181	4.6140*e* − 005	0.0037	0.0026	0.0017
IS-MOEA	Mean	0.8716	0.8518	0.9670	0.7838	0.7444	0.8472
	Var.	0.0115	0.0088	3.899*e* − 004	0.0208	0.0496	0.0127

**Table 6:** Distribution of HV value using Wilcoxon rank-sum test.

	OSY	TNK	CONSTR	CTP1	CTP2	CTP3
HV(CMBOA, NSGA-II)	2.5721*e* − 007	3.0199*e* − 011	**0.0555** **>0.05**	4.6159*e* − 010	**0.0824** **>0.05**	8.9934*e* − 011
HV(CMBOA, IS-MOEA)	1.4110*e* − 009	3.0199*e* − 011	2.8314*e* − 008	3.3242*e* − 006	**0.3255** **>0.05**	9.8329*e* − 008

	CTP4	CTP5	CF1	CF2	CF4	CF6

HV(CMBOA, NSGA-II)	4.0772*e* − 011	1.0937*e* − 010	2.6099*e* − 010	0.0044	**0.8534** **>0.05**	5.9706*e* − 005
HV(CMBOA, IS-MOEA)	3.3384*e* − 011	4.5726*e* − 009	3.0199*e* − 011	1.0937*e* − 010	4.1825*e* − 009	3.0199*e* − 011
